# Emotion Regulation Difficulties, Financial Anxiety, and Trading Motivation in Cryptocurrency Users

**DOI:** 10.1192/j.eurpsy.2026.10482

**Published:** 2026-07-31

**Authors:** H. O. Sonkurt, T. Gündüz

**Affiliations:** Psychiatry, Eskişehir Osmangazi University, Eskişehir, Türkiye

## Abstract

**Introduction:**

Cryptocurrencies have rapidly become significant for financial participants. Their decentralized structure, constant availability, and high volatility create a high-risk, high-reward environment that draws increasing attention to the psychology of traders.

While research has linked cryptocurrency investing to impulsivity, risk-taking, and gambling-like behaviors, the role of emotion regulation remains overlooked. Psychopathology literature highlights emotion regulation difficulties as transdiagnostic across depression, anxiety, and addictions, and as crucial in decision-making under uncertainty. Financial choices are shaped not only by cognition but also by emotion, underscoring the importance of this perspective.

**Objectives:**

This study examined the relationships among emotion regulation skills, financial anxiety, and trading motivations in cryptocurrency users, and their predictive role in pathological trading, a phenomenon that resembles gambling behavior. We examined whether pathological traders show greater emotion regulation difficulties and financial anxiety than non-pathological traders; how trading motives relate to distress; whether higher difficulties and anxiety predict frequent trading; and whether a brief index could identify high-risk investors.

**Methods:**

Ethical approval was obtained on July 18, 2025. Participants were 302 active traders aged 18+. The online survey included a sociodemographic form, the Problematic Cryptocurrency Trading Scale, Difficulties in Emotion Regulation Scale–16, Patient Health Questionnaire-4, Financial Anxiety Scale, and Gambling Motives Questionnaire. Three attention checks were embedded. The survey was distributed via Turkish crypto communities (via Twitter, Reddit, Telegram). Informed consent was obtained and data were anonymous. Collected data were analyzed with the Jamovi program, with descriptive statistics, correlations, and comparative tests.

**Results:**

Two distinct forms of problematic crypto use—Withdrawal & Tolerance and Money Seeking & Denial—were linked to different predictors. Withdrawal & Tolerance was strongly associated with impulsivity, avoidance and monetary motives, margin trading, and frequent checking. Money Seeking & Denial showed weaker associations, with avoidance motives as the strongest predictor. Regression models confirmed these patterns: Withdrawal & Tolerance was best predicted by impulsivity, gambling motives, margin trading, and price-tracking frequency (explaining >50% of variance), while Money Seeking & Denial was predicted mainly by avoidance motives, with modest input from impulsivity.

**Conclusions:**

Problematic crypto use reflects distinct psychological pathways: Withdrawal & Tolerance is driven by impulsivity, gambling-related motives, and trading behaviors, whereas Money Seeking & Denial is primarily linked to avoidance motives. Tailored interventions should address both emotion regulation and motivational processes.

**Disclosure of Interest:**

None Declared

